# TLR-Dependent Human Mucosal Epithelial Cell Responses to Microbial Pathogens

**DOI:** 10.3389/fimmu.2014.00386

**Published:** 2014-08-12

**Authors:** Ryan McClure, Paola Massari

**Affiliations:** ^1^Department of Microbiology, Boston University School of Medicine, Boston, MA, USA; ^2^Section of Infectious Diseases, Department of Medicine, Boston University School of Medicine, Boston, MA, USA

**Keywords:** epithelial cells, mucosal tissues, pattern recognition receptors, immunity, bacteria

## Abstract

Toll-like receptor (TLR) signaling represents one of the best studied pathways to implement defense mechanisms against invading microbes in human being as well as in animals. TLRs respond to specific microbial ligands and to danger signals produced by the host during infection, and initiate downstream cascades that activate both innate and adaptive immunity. TLRs are expressed by professional immune cells and by the large majority of non-hematopoietic cells, including epithelial cells. In epithelial tissues, TLR functions are particularly important because these sites are constantly exposed to microorganisms, due to their location at the host interface with the environment. While at these sites specific defense mechanisms and inflammatory responses are initiated via TLR signaling against pathogens, suppression or lack of TLR activation is also observed in response to the commensal microbiota. The mechanisms by which TLR signaling is regulated in mucosal epithelial cells include differential expression and levels of TLRs (and their signaling partners), their cellular localization and positioning within the tissue in a fashion that favors responses to pathogens while dampening responses to commensals and maintaining tissue homeostasis in physiologic conditions. In this review, the expression and activation of TLRs in mucosal epithelial cells of several sites of the human body are examined. Specifically, the oral cavity, the ear canal and eye, the airways, the gut, and the reproductive tract are discussed, along with how site-specific host defense mechanisms are implemented via TLR signaling.

## Introduction

All organisms have some form of protective mechanisms against pathogens. In many instances, innate immunity functions are the first, and sometimes, the only barrier to infection by invading organisms. In human beings, innate immunity is not only mediated by professional immune cells, but also by non-professional cell types that contribute to defense responses by secreting substances with anti-microbial activity and inflammatory mediators that favor rapid and direct involvement of professional immune cells. Many, if not all, of these responses are dependent on detection of invading microorganisms. The toll-like receptors (TLRs) family is one of the best characterized among several cellular effectors for pathogen detection (1). TLRs are a family of trans-membrane proteins widely expressed by eukaryotic cells and recognize ligands that are present in virtually all types of microorganisms. Once binding takes place, activation of signaling pathways downstream of TLRs plays a major role in directing both innate and adaptive host immune responses. Thus, TLRs represent one of the first and most important lines of defense against bacterial, viral and fungal pathogens and parasites that may interact with and harm the human host. However, two major aspects relative to TLR-dependent pathogen recognition and subsequent responses need to be carefully considered: most microorganisms that colonize the human host are not pathogens, and, depending on the site of colonization/infection, different defense responses may be necessary or appropriate to counteract such infections. This review explores how TLR signaling is regulated in mucosal epithelial cells to mediate specific host responses to commensal or pathogenic microbial infections. Such control is exerted via a number of mechanisms, including regulation of receptor expression levels, cellular localization (i.e. cytosolic or surface expression) and positioning within the tissue (apical or basolateral expression), and also depending on the tissue body site.

## Toll-Like Receptors: Overview of Structure and Signaling Pathways

Toll-like receptors were discovered almost two decades ago and their importance in regulation of immune responses was immediately recognized, enhancing our understanding of many phenomena that define host innate and adaptive immunity. TLRs recognize microbial and viral products with specific structural features. Such products are classified as pathogen-associated molecular patterns (PAMPs) (2). As many microorganisms colonize the human host without causing disease, the term CAMPs has been introduced for commensal-associated molecular patterns (or the more generic term MAMPs, for microbial-associated molecular patterns) that are also recognized by TLRs (3). In addition, endogenous ligands that induce inflammation in the absence of infection can also activate TLR-dependent signaling and are defined as danger-associated molecular patterns (DAMPs) (4).

Toll-like receptors are trans-membrane proteins that contain a horseshoe-shaped extracellular or cytoplasmic leucine-rich repeat (LRR) domain and an intra-cytoplasmic toll/IL-1R (TIR) domain [homologous to the corresponding intracellular domain of the IL-1 receptor (IL-1R)], which are connected by a single trans-membrane domain. The LRR domain is responsible for ligand recognition and the TIR domain for intracellular signal transfer.

**Figure 1:**
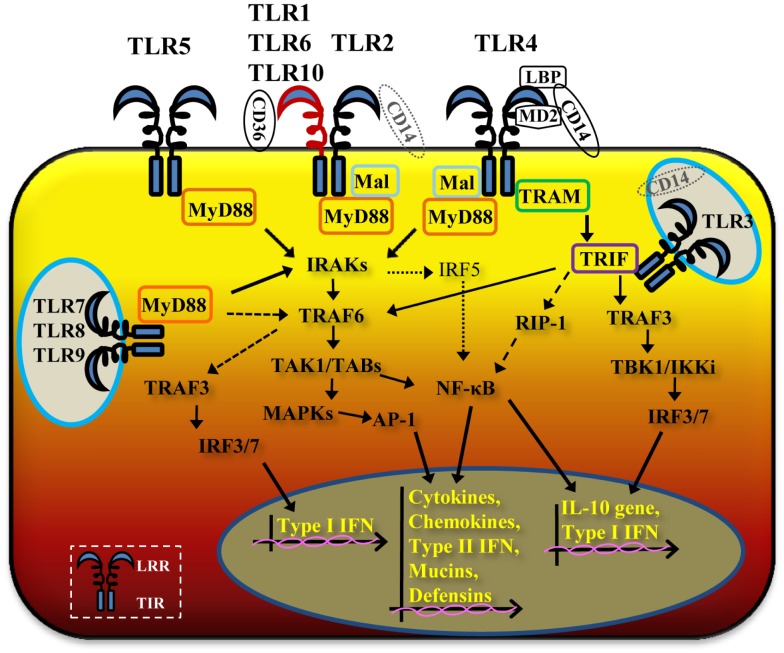
**Schematic cartoon of TLR signaling pathways**. TLR2/TLR1 and TLR2/TLR6 heterodimers and TLR4 and TLR5 homodimers are located on the cell surface, while TLR3, TLR7, TLR8 and TLR9 homodimers have an intracellular localization. Occasionally, in specific epithelial cell types and tissues, TLR4 can be expressed intracellularly or TLR3 and TLR9 on the cell surface. Ligand binding to the leucine-rich repeat (LRR) domain of TLR dimers brings the TIR domains of adjacent TLRs in proximity, allowing multiple signaling pathways via different adaptor molecules. TLR2/TLR1 and TLR3 potentially cooperate with CD14, TLR2/TLR6 with CD36 and TLR4 requires LBP, CD14 and MD-2. TLR1, TLR2, TLR4, TLR5, TLR6, TLR7, TLR8 and TLR9 activate the MyD88-dependent pathway, with cooperation of Mal for TLR2 and TLR4. Through MyD88, IRAKs and TRAF6, TLR2/TLR1 and TLR2/TLR6, TLR4, TLR5, TLR7, TLR8, and TLR9 signaling pathways lead to activation of NF-κB and MAPKs, with production of inflammatory cytokines and chemokines, type-II interferon, mucins, and defensins (solid thick arrows). TLR7, TLR8, and TLR9 also induce MyD88-dependent activation of TRAF6 and TRAF3 (dashed arrows). TLR3 and TLR4 activate a MyD88-independent pathway via TRIF and, in cooperation with TRAM for TLR4 (solid medium arrows), also leading to NF-κB and MAPKs activation and of inflammatory mediators, type-II interferon, mucins, and defensins.The MyD88-independent pathway also induces IRF3 and IRF7 activation, with production of type-I IFNs and IL-10 gene activation.

In humans 10 TLRs have been identified to date and comprise both extracellular and intracellular receptors (1). TLR1, TLR2, TLR4, TLR5, TLR6, and TLR10 are surface-expressed and recognize extracellular microorganisms and ligands. TLR3, TLR7, TLR8, and TLR9 are intracellular, localizing into cytosolic endosomal compartments via a UNC-93B-assisted translocation mechanism (5), and are engaged by microorganisms and ligands that have already crossed the cell membrane barrier. In some instances, intracellular TLRs, such as TLR3 and TLR9, can be expressed on the cell surface, and extracellular TLRs, such as TLR4, can also have an intracellular localization, depending on the cell type (6, 7). For all TLRs, ligand binding to the LRR domain induces formation of receptor homodimers or, in some cases, heterodimers (Figure 1). A resulting TIR domain conformational change allows interactions between TIR domains of adjacent TLRs and binding of additional adaptor proteins essential for triggering intracellular signaling cascades. Adaptor proteins identified to date include the myeloid differentiation factor 88 (MyD88) (8), the MyD88 adaptor-like (Mal) (9) [also called TIR domain-containing adaptor protein, TIRAP (10)], the TIR domain-containing adaptor protein inducing interferon-β (TRIF) (11) [also called TIR-containing adaptor molecule, TICAM (12)], and the TRIF-related adaptor molecule (TRAM) (13). TLR signaling is also subject to negative regulation by a variety of inhibitory factors, including the Toll-interacting protein (Tollip), IRAK-M, the sterile α- and HEAT-Armadillo-motif-containing protein (SARM), and the B cell adaptor for PI3K (BCAP) (14), which inhibit downstream steps in the TLR-dependent signaling cascades. The crystal structure of several TLRs has been solved, either alone or in complex with ligands (15–19), expanding our understanding of the molecular mechanisms of TLR activation and of the co-factors that are required for signaling.

With the exception of TLR3, all TLRs require MyD88 recruitment to the TIR domain. TLR2 and TLR4 signaling require not only MyD88 but also the cooperation of Mal/TIRAP (Figure 1). Through MyD88, members of the IL-1R-associated protein kinases (IRAKs) IRAK4, IRAK1, and IRAK2 are activated (20). This is directly followed by activation of the tumor necrosis factor receptor-associated factor 6 (TRAF6) (21) and RIP (22), which proceed to activate a complex made of TGF-β-activated kinase 1(TAK1) and TAK1-binding proteins (TAB1, TAB2, and TAB3). Lastly, gene expression regulatory factors of the MAPK family (ERK, JNK, p38) and NF-κB are activated (Figure 1), inducing cell survival and proliferation, immune cell activation, production of pro-/anti-inflammatory mediators (cytokines and chemokines), interferons, and anti-microbial products. Activation of intracellular TLR7, TLR8, and TLR9 also proceeds via MyD88, but can trigger TRAF6, IRAK4, and TRAF3-dependent activation of IRF7, which translocates to the nucleus and induces production of type-I interferon (13, 23) (Figure 1).

A MyD88-independent pathway is triggered by TLR3 and TLR4 (in addition to the TLR4/MyD88-dependent signaling pathway) and, potentially, by TLR2 (24, 25). The TLR3 MyD88-independent pathway is mediated by TRIF and the TNF receptor associated factor protein TRAF3, inducing non-canonical IKKs, TBK1, and IKKε pathways, activation of IRF3, and secretion of type-I IFN (IFN-β) and IL-10 (26) (Figure 1). TLR3-TRIF signaling can also drive activation of MyD88-dependent downstream components TRAF6 and RIP1, thus converging on activation of NF-κB (Figure 1). The TLR4/MyD88-independent pathway leads to recruitment of TRIF via activation of TRAM and downstream segregation of cell activation via both TRAF6/RIP1 and TRAF3/IRF3 pathways (27) (Figure 1).

## TLR Ligands: Overview of Interactions

Toll-like receptors recognize a large variety of structurally defined, but not necessarily structurally related ligands. TLR3, TLR5, TLR7, TLR8, and TLR9 recognize “unique type” of ligands. TLR3 recognizes viral double-strand RNA (dsRNA) and synthetic analogs of dsRNA, such as Poly I:C (28), with the potential contribution of the adaptor molecule CD14 (29). Bacterial flagellin is the ligand for TLR5 (30), TLR7 and TLR8 recognize viral single-strand RNA, miRNA and the anti-viral compounds, imidazoquinolines (31–34), and TLR9 recognizes unmethylated CpG DNA of bacterial and viral origin (35) as well as the malaria pigment, hemozoin [likely due to its being coated with malarial DNA (36)]. A unique ligand for TLR10 is currently unknown but this receptor is thought to heterodimerize with TLR2 and share recognition of ligands that are in common with those that are recognized by TLR1. Recently, a role for TLR10 has been suggested in responses to pathogen infections (37, 38), Crohn’s disease (39), and even cancer (40).

Ligand discrimination by TLR2 and TLR4 is a complex process that is not only dependent on ligand compatibility but also requires the presence of specific co-receptors and accessory molecules. In the case of TLR4, recognition of its best characterized ligand, bacterial lipopolysaccharide (LPS) occurs when this is in complex with the accessory molecules lipid binding protein (LBP) and the lipid A binding protein (CD14), and is presented to TLR4 in the presence of the myeloid differentiation protein 2 (MD-2) (41). Homodimerization of the TLR4/MD-2 complex following ligand binding then brings the TLR4 TIR domains in close proximity, which proceeds to trigger signaling cascades via the MyD88-dependent or the MyD88-independent pathways (Figure 1). It has been suggested that this dichotomy may be influenced by the LPS type (smooth vs. rough or lipid A). In the presence of CD14, all LPS types can induce TLR4 activation via both pathways. In the absence of CD14, smooth LPS fails to induce TLR4 activation, while lipid A induces signaling via Mal/MyD88 (42). The details of the molecular interactions of TLR4 with its accessory molecules and ligands have been elucidated in elegant crystal structure studies (17). A novel factor involved in TLR4-mediated signaling has been described, the TLR4-interactor with LRRs, TRIL, which is highly expressed particularly in the brain and enhances TLR4-dependent signaling by LPS (43). Some types of bacterial LPS signal via TLR2, for example *Porphyromonas gingivalis* LPS, although some components of TLR4-dependent signaling are also involved (44). In addition to LPS, TLR4 also recognizes viral components and endogenous ligands, such as β-defensin 2 (45), high mobility group box 1 protein (HMGB1) (46), fibronectin extra domain A (F-EDA), heat shock proteins and other molecules (47), although the contribution of contaminating LPS to the effect of some of these molecules is still unclear.

An even more complex picture characterizes signaling via TLR2, which can form heterodimers with either TLR1 or TLR6. TLR2 recognizes a broad range of ligands with very different structural features. The first described TLR2 ligands are lipopeptides and lipoproteins, shown to engage TLR2/TLR1 or TLR2/TLR6 heterodimers depending on different acyl group patterns. The synthetic triacylated lipoprotein Pam_3_CSK_4_ is a specific ligand for the TLR2/TLR1 dimer (48) while the diacylated lipoprotein Pam_2_CSK_4_ binds to and signals via the TLR2/TLR6 dimer (but can also function via TLR2/TLR1) (49, 50). The molecular and structural details of the TLR2/TLR1- and TLR2/TLR6-ligand complexes have been elucidated by co-crystallization studies that have paved the way in defining these sophisticated interactions and the role of the accessory molecules CD14, LBP, and CD36 in ligand-driven complex formation (16, 51, 52). Other bacterial ligands for TLR2 include cell wall components such as lipoteichoic acid (LTA) (53), glycolipids, lipoarabinomannan (54), β-glucans (55) and zymosan (56). TLR2 activation by peptidoglycan (PG) is controversial and this molecule is also known to signal via another intracellular pattern recognition receptor, Nod2, a member of the nucleotide oligomerization domain (NOD)-like receptors (NLRs) family (57–59). In addition, bacterial proteins of diverse origin with no structural similarities and no lipid components have also been shown to activate cells via TLR2 signaling, for example porins and toxins. Porins from *Neisseriae* species, *Fusobacterium nucleatum* and *Chlamydia* induce TLR2/TLR1-dependent signaling (60–64), while *Shigella* and *Salmonella* porins induce signaling via TLR2/TLR6 (65, 66). *Haemophilus* porin is also considered a TLR2 ligand (67). Other well-described TLR2 protein ligands are the pentameric B subunit of the *Escherichia coli* type-II heat-labile enterotoxin [LT-IIa-B(5) and LT-IIb-B(5)] (68), bacterial fimbriae (69) and the PPE18 protein from *Mycobacterium tuberculosis* (70). Furthermore, endogenous ligands and DAMPs are also associated with TLR2-dependent signaling, including HSPs, HMGB1, uric acid, fibronectin and other extracellular matrix proteins, and some types of LPS, as discussed in the previous section.

## TLRs in Human Epithelial Cells: Overview of Expression and Functions

**Figure 2:**
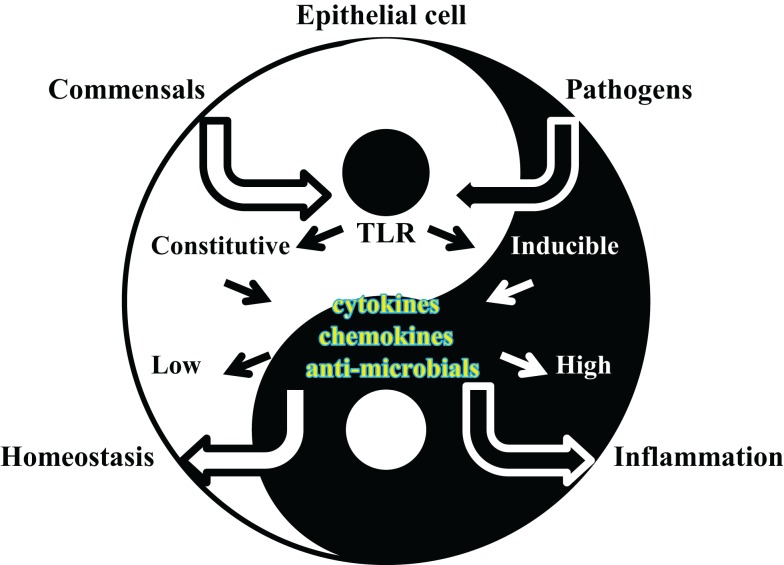
**Cartoon of epithelial cell/microorganisms dynamic interactions**. Bacterial recognition by TLRs expressed by epithelial cells leads to activation of local defense responses in the epithelial tissues that become colonized. TLR-dependent induction of anti-microbial substances and inflammatory mediators contribute to bacterial clearance by controlling organism survival and by triggering host local and systemic immune responses, respectively. While such processes are pivotal against pathogens, they are not desirable against the local commensal microflora. In a Yin–Yang balance of epithelial tissue homeostasis and defense/inflammatory responses, regulation of TLR signaling is crucial for inducing appropriate cell responses to microorganisms of different nature.

In humans TLR expression is nearly ubiquitous in immune cells, where it drives innate and adaptive immune mechanisms such as activation of antigen-presenting cells (APCs), secretion of inflammatory mediators, T cell differentiation and antibody production. By contrast, TLR expression is less widespread in cells of non-hematopoietic origin, such as epithelial cells (Table 1). Since TLRs are specialized in recognition of microbial products, it appears reasonable that they have evolved to be localized at the best potential host/microbe interface for a rapid initial response. For example, depending on the cell type and the body location, TLR protein expression may not be detected despite the presence of TLR mRNA, extracellular TLRs may present an intracellular localization in endosomal compartments (i.e., TLR4) while intracellular TLRs can be found on the cell surface (i.e., TLR3 or TLR9), and selected TLRs can be expressed in a tissue-specific manner. TLR-dependent activation of immune responses by a pathogen is indiscriminately triggered in APCs, but a similar modality of activation of epithelial cells may lead to unnecessary responses to the large number of commensal organisms found throughout the majority of non-sterile body surfaces that are in constant contact with the environment (Figure 2). The best and most studied examples include the selective expression of TLR2 and TLR4 by cells of mucosal epithelial sites such as the oral cavity, the upper and lower airways (including the nasal passage), the ear and the eye, the gut and the reproductive tract, as well as the skin (even if the majority of the skin tissue comprises cells of non-mucosal nature). Although the first function of these cells is that of offering a mechanical barrier against pathogens, they also have an intimate relationship with both circulating and local immune cells [i.e., resident neutrophils, dendritic cells (DCs) and macrophages].

**Table 1:** **TLR mRNA and protein expression in mucosal epithelial cells**.

Tissue	TLR	
	mRNA	Protein
**ORAL EPITHELIA**		
Gingival	TLR1 (71, 72), TLR2 (73–75), TLR3 (73), TLR4 (73, 75, 76), TLR5 (71, 72), TLR6 (71, 72), TLR7 (73), TLR8 (71–73), TLR9 (71, 72)	TLR1 (71), TLR2 (71–73, 75–78), TLR3 (71, 73, 78), TLR4 (71–76), TLR5 (71, 77, 78), TLR6 (71, 72, 78), TLR7 (71, 73), TLR8 (71), TLR9 (71, 74, 75, 79)
Salivary	TLR1–TLR10 (80, 81)	TLR1–TLR4, TLR7 (80)
Tonsillar	TLR1–TLR6, TLR9, TLR10 (80, 82)	TLR2, TLR3 (82)
Ear epithelia	TLR2–TLR4, TLR9 (83–86)	TLR2–TLR4, TLR9 (83–86)
**OCULAR EPITHELIA**		
Corneal	TLR1 (87), TLR2 (6, 87–90), TLR3 (87–89), TLR4 (6, 87–89), TLR5 (87, 91), TLR6 (87), TLR7 (87, 88), TLR9 (87–89), TLR10 (87)	TLR1 (92), TLR2 (6, 87, 90, 92–96), TLR3 (6, 87–89, 92, 93), TLR4 (6, 88, 90, 93, 96–98), TLR5 (87, 91–95, 97), TLR6 (92), TLR9 (87, 89)
Conjunctival	TLR1 (87), TLR2, TLR3 (87, 88), TLR4 (87, 88, 99), TLR7 (87, 88), TLR9 (87, 88, 99), TLR10 (87)	TLR3 (88), TLR4 (88, 99), TLR9 (99)
Retinal	TLR1–TLR7, TLR9 (100)	TLR2–TLR4 (100)
Iris	TLR4 (98)	TLR4 (98)
**AIRWAY EPITHELIA**		
Nasal	TLR1–TLR10 (101, 102)	TLR2 (102, 103), TLR3 (102), TLR4 (103)
Tracheal/bronchial	TLR1 (7, 81, 104), TLR2 (7, 81, 104, 105), TLR3 (7, 81, 104), TLR4 (7, 81, 104, 106), TLR5–TLR10 (7, 81, 104)	TLR1, TLR2 (7, 104, 105, 107), TLR3 (7, 104, 107), TLR4 (7, 104, 106, 107), TLR5, TLR6 (7, 104, 107), TLR7, TLR9, TLR10 (7)
Lung	TLR1 (81, 108), TLR2 (81, 108–111), TLR4 (81, 101, 103, 109, 111, 112), TLR5, TLR6 (81, 108)	TLR2 (105, 109, 110), TLR4 (103, 109–112), TLR5 (108)
**GUT EPITHELIA**		
Esophageal	TLR1–TLR5 (113)	TLR1–TLR3 (113), TLR4 (113, 114), TLR5 (113)
Gastric	TLR2, TLR4, TLR5 (115–117)	TLR2, TLR4, TLR5 (115–117)
Intestinal	TLR1 (81), TLR2 (81, 118, 119), TLR3 (81), TLR4 (81, 118, 120–122), TLR5–TLR10 (81)	TLR2 (81, 118, 119) TLR3 (123), TLR4 (118, 120–122), TLR5 (119, 123), TLR9 (123)
M cells/Paneth cells		TLR2, TLR4, TLR5 (124)
**GENITO-URINARY EPITHELIA**		
Male
Penile, urethra testis, prostate	TLR1, TLR2 (81), TLR3 (81, 125–127), TLR4–TLR7 (81), TLR8 (81, 125–127) TLR9, TLR10 (81)	TLR2 (81, 125–128), TLR3, TLR4 (128), TLR8 (125), TLR9 (81, 125–127)
Female
Vagina	TLR1–TLR6, TLR9, TLR10 (129, 130)	TLR1 (129, 130), TLR2 (129–131), TLR3, TLR5, TLR6 (129–131)
Endocervix/endocervix	TLR1–TLR3, TLR5–TLR9 (130, 132)	TLR1- TLR3, TLR5, TLR6, TLR9 (130, 132)
Endometrium, uterus/fallopian tubes	TLR1–TLR6 (130), TLR7–TLR9 (130, 133, 134)	TLR1, TLR2 (130), TLR3 (130, 134), TLR4–TLR6 (130), TLR7–TLR9 (130, 132–134)
Urinary tract/renal	TLR1–TLR5, TLR9 (135, 136)	TLR2–TLR4 (135, 137–140), TLR5, TLR9 (137)

Direct defense functions mediated by TLR signaling in mucosal epithelial tissues include induction of anti-microbial substances and other soluble mediators for local and systemic control of infections. Production of anti-microbial substances is beneficial for controlling mucosal epithelial tissue colonization/infection but production of inflammatory mediators is a double-edged sword. In fact, while pro-inflammatory cytokines trigger recruitment and activation of APCs at the site of infection, they may also favor inflammatory tissue damage. One example is the damaging effect of TNF-α on oral bone integrity. Following infection by oral pathogens such as *P. gingivalis*, gingival epithelial cells, and macrophages that are recruited to this site secrete high levels of TNF-α, which then causes enhanced inflammatory oral bone loss. In addition, uncontrolled secretion of inflammatory cytokines is not desirable for maintaining local tissue homeostasis in the presence of the commensal microbiota. On the other hand, lack of inflammatory responses is similarly dangerous in the instances when pathogen infections or commensal imbalance may occur.

The inflammatory cytokines and chemokines most frequently produced by epithelial cells via TLR stimulation include those directly involved in inflammatory and immune regulation (i.e., IL-1α and IL-1β, IL-6, IL-10, IL-13, TNF-α, and TGF-β), those with chemotactic effects (such as IL-8, MCP-1, MIP-1, and RANTES) and growth and differentiation factors (i.e., IL-3, IL-7, G-CSF, and GM-CSF). The activity of some of these mediators encompasses several of the categories mentioned above. However, some promote inflammation and amplify immune responses, for example IL-1β, IL-8, RANTES, or TNF-α, while others dampen such responses, such as IL-10, IL-37, and TGF-β (141). Besides secreting inflammatory mediators, epithelial cells influence mucosal innate and adaptive immunity by also producing factors that directly affect DC, B and T cell functions, such as the B cell-activating factor of the TNF family (BAFF), a proliferation-inducing ligand (APRIL) (142, 143) and type-I interferons.

## TLR Expression and Responses in Human Mucosal Epithelial Tissues

### Oral epithelium

The gingival epithelium is composed of a variety of cell types including keratinized and non-keratinized, stratified and flat squamous cells, which are exposed to an enormous number of microorganisms of both commensal and pathogenic nature. It is thought that up to 10^10^ bacteria can be found in the oral cavity. In physiologic conditions, a relatively small number of resident immune cells, including neutrophils, lymphocytes and monocytes/macrophages, are located within the oral cavity epithelial tissue. Upon oral pathogen infection, TLR-dependent gingival inflammation causes an influx of neutrophils, monocytes and lymphocytes to facilitate bacterial clearance (144).

Toll-like receptor expression and functions in the oral cavity are very important for the maintenance of oral tissue homeostasis because of the constant presence of commensal microbes (Table 1). Expression of mRNA for TLR1 to TLR9 has been detected in oral epithelial cells (including the tongue), although actual TLR protein expression and cellular localization can be variable and inducible. TLR2 is highly expressed in cells of the gingival basal layer but lower levels are observed in cells of the superficial layers, more exposed to the environment and to microorganisms. While an opposite spatial relationship may be expected to insure recognition of colonizing microorganisms, this is a strategy that facilitates TLR-dependent inflammatory response only when invading pathogens are detected in the basal cell layer. A similar pattern is observed for TLR1, TLR3, TLR4, TLR5, and TLR9 expression, also depending on the state of the tissue (inflamed vs. non-inflamed) (71, 72). TLR7 and TLR8 expression is comparable in both healthy and infected tissues. In addition, acute and persistent gingival inflammation also enhances the expression of TLR2 and TLR4, favoring downstream local innate immune responses (73). In chronic oral inflammatory conditions (i.e. bacterial periodontitis or other pathologies), TLR4 expression in the gingival epithelium decreases, likely to dampen inflammatory responses that may exacerbate damage to oral tissue and bone (76). Variable levels of TLR expression have also been observed as a consequence of other oral chronic inflammatory conditions, for example caused by lichen planus. In this case, increased TLR4 and TLR9 protein expression and decreased TLR2 mRNA have been detected (74, 75). High constitutive expression of TLR1, TLR2, TLR3, TLR4, and TLR7 mRNA has also been shown *in vitro* in salivary gland epithelial cells, with TLR3 protein levels being the highest (80). mRNA for all TLRs except TLR7 and TLR8 has been observed in tonsillar epithelial cells at the junction between the oral cavity and the airways. A strong expression of TLR2 and TLR3 mRNA is observed in both tonsillar cell lines and primary cells, and detection of actual TLR2 and TLR4 proteins and their activity appears variable (82).

The likely most relevant defense mechanism that is mediated by TLR signaling in the oral cavity is induction of anti-microbial substances such as defensins (α-, β-, and θ-type). Human β-defensin (hBD)-1 to hBD-4 mRNA and proteins are expressed in the oral epithelium (145). hBD-1 is constitutively expressed, hBD-2 and hBD-3 are inducible in the basal layer epithelial cells via TLR2, TLR3, TLR4, TLR5 and TLR9 signaling, and by general inflammatory conditions of the gingival epithelium (i.e., in the presence of IL-1β and TNF-α). hBD-4 is only induced by bacterial infections (146). In a feedback mechanism, β-defensins also induce TLR signaling and recruitment/activation of immature DCs, monocytes and memory T cells in the oral epithelium, which thus places these substances at a cross-road between an immune effector and an immune inducer produced by epithelial cells (147). Overall, activation of TLR2 signaling is generally more frequent than that of TLR4 in gingival epithelial cells, inducing a strong activation of MAPKs and NF-κB pathways than controls production of antibacterial substances (148).

Oral epithelial cells do not generally secrete high levels of inflammatory mediators, likely to avoid excessive local innate immune responses resulting in tissue destruction. Secretion of IL-8 in response to TLR9, TLR2 and TLR5 stimulation and, to a lesser extent, to TLR4 stimulation has been shown in gingival epithelial cells, which is enhanced by a prior cell exposure to IFN-γ (77, 79, 149). Besides IL-8, secretion of other inflammatory cytokines directly involved in innate immunity, such as IL-1β and TNF-α, as well as that of APCs chemo-attractants has been shown. Thymic stromal lymphopoietin (TSLP) is also expressed by oral epithelial cells following TLR3, TLR5 and TLR2/TLR6 activation (78).

### Ear epithelia

Toll-like receptor expression has been detected in the ear epithelium (Table 1), indicating that this tissue is suited to respond to pathogens and initiate immune and defense responses accordingly. Primary epithelial cells of the human ear and middle ear/inner ear epithelial cell lines express functional TLR2 but not TLR4, shown by their responsiveness to *Haemophilus influenzae* whole cell lysates stimulation *in vitro* and up-regulation of defensins mRNA expression, but not to purified LPS (83). TLR2-dependent stimulation is inhibited by anti-TLR2 blocking antibodies (84). From immunohistochemistry studies of biopsies from the normal ear canal and acquired cholesteatoma (an abnormal growth of keratinized squamous epithelium), it appears that expression of TLR2, TLR3 and TLR4 is detected and regulated as a function of cholesteatoma (85). Studies on TLR expression and function in animal models support expression of TLR2, TLR4, and TLR9 in auditory cells (86).

### Ocular epithelia

A larger number of studies exist regarding TLR expression in the human eye (93) as compared to the ear (Table 1). Expression of mRNA for TLR1, TLR2, TLR3, TLR4, TLR5, TLR6 and TLR9 has been shown in human corneal epithelial cells (88), where extracellular TLR3 and intracellular TLR2 and TLR4 expression can be detected (6). TLR2 and TLR5 functionality in these cells has been assessed by loss-of-activity using anti-TLR2 and -TLR5 antibodies. It is not clear whether lack of TLR4 or MD-2 protein expression is involved in un-responsiveness to TLR4 ligands (97). Corneal epithelial cells respond to TLR stimulation mostly by secreting defensins and by further regulating expression of TLR mRNA (87, 92, 94, 95). In retinal epithelial cells, TLR2, TLR3, and TLR4 mRNA expression has been detected and is regulated by signaling via TLR3, which induces high levels of TLR3 and TLR9 protein expression (89, 100). Induction of interferons, IL-8 and MCP-1 can be inhibited by anti-TLR3 antibodies, but stimulation via TLR9 only induces IL-8 secretion. mRNA for TLR1, TLR6, TLR7 and TLR9 and TLR4 protein expression has also been detected in section of whole human eyes and iris epithelial cells (98). Lastly, TLR9 is expressed in conjunctival epithelial cells and its expression is regulated by nerve growth factors (99). In the human eye, the major function of TLR-dependent signaling is induction of anti-microbial substances. While hBD-1 to hBD-4 are expressed constitutively, hBD-2 expression is increased by TLR2 and TLR5 stimulation for example, in a *Pseudomonas aeruginosa* infection model of corneal epithelial cells (90, 95). In the ocular epithelial tissue, expression of hBD-9 is also induced by TLR2, TLR3, TLR4 and TLR5 signaling (150, 151). In immortalized human eye tissue, mRNA expression and secreted IL-1β, IL-6, IL-8, MCP-1 and sICAM-1, IL-32, IL-33 and TNF-α are induced by TLR2, TLR3, TLR4 and TLR5 signaling in response to viral and bacterial stimulation (91, 94, 96, 152).

### Airway epithelium

Crucial regulation of TLR expression is well-documented in the airways (Table 1), where it critically influences airway defense mechanisms and local immune responses. While expression of TLR mRNAs is generally detected in respiratory tract epithelial cells, protein expression often varies, depending on tissue site and on host physiologic vs. disease condition (i.e., normal vs. inflammation or allergy) (7, 104). The most relevant TLRs in the airways epithelia are TLR2 and TLR4. Their expression is maintained at low levels and preferentially on the basal cell layers. Following infection by pathogens and in inflamed tissues, increased expression of TLR2 and TLR4 has been reported (101, 109, 110). TLR4 is often intracellular, initially located in the Golgi complex, but is readily transferred to the cell surface for pathogen recognition (112). TLR2 and TLR4 responses in the airway epithelium are also influenced by the constitutively low or absent expression of MD-2 and CD36 (106, 153) and by negative TLR regulatory factors such as IRAK-M and Tollip, which dampen cell activation in physiologic conditions. Exposure to TLR4 ligands, such as killed *H. influenzae* or its purified P6 outer membrane protein, to TNF-α and IFN-γ can induce MD-2 expression and cell responsiveness. TLR3 and TLR5 also play important roles in the human airway epithelia by recognizing viral and bacterial ligands (107, 108).

Toll-like receptor-dependent activation of airway epithelial cells can induce different responses, depending on the tissue location along the respiratory tract. The upper respiratory epithelium, which includes the nasal cavity, pharynx, and the larynx, is generally in contact with a high number of (mostly commensal) organisms [up to 10^7^organisms/nostril and up to 10^8^ organisms in the nasopharynx (154)]. The first and most common defense response in these epithelia is production of mucus. Expression of mucins, the major protein components of mucus, is induced directly, by TLR signaling and indirectly, by high levels of IL-8 and TNF-α induced via TLRs (155). In turn, mucins can also further regulate TLR signaling (156). In epithelial cells of the nasal mucosa, TLR signaling induces production of anti-microbial substances including hBD-1 to hBD-4, relevant for defense mechanism in both disease conditions and allergy (103). In the lower respiratory tract, which includes the trachea, primary bronchi, and the lungs, TLR-dependent responses are also regulated through expression levels and localization mechanisms. These epithelial tissues are relatively sterile but can become exposed to microorganisms descending from the upper respiratory tract. Studies of human lung epithelial tissues have shown TLR4 expression and signaling in response to bacterial LPS (112) and in disease conditions, such as chronic obstructive pulmonary disorder (COPD), asthma and allergy (103, 111). In tracheal epithelial cells, TLR3 expression on the luminal and basal side, and TLR2, TLR6 and TLR1 basolateral expression, have been reported. Low levels of TLR2, TLR4, TLR5, TLR7, TLR9 and TLR10, and high levels of TLR6 are shown, and TLR3, TLR7, and TLR9 are present in both intracellular compartments and on the cell surface (7). Also in lower respiratory tract, TLR signaling induces production of anti-microbial substances. HBD-1 is constitutively expressed in these epithelia, hBD-5 and hBD-6 are not expressed and increased expression of hBD-2 to hBD-4 is observed in a TLR-dependent manner (105, 106, 157). Besides defensins, other antibacterial molecules are induced by TLR signaling, such as lysozyme, nitric oxide (NO), and LL-37 (158). TLR signaling also leads to production of cytokines. Soluble inflammatory mediators in the upper and lower respiratory epithelia are desirable for promoting local recruitment of professional phagocytic cells that participate in pathogen clearance. Accordingly, TLR-dependent secretion of TNF-α, IL-8, MIP-1α, MIP-1β, RANTES, GRO-α, -β, and -γ, IL-6, IL-5, and TGF-β promotes an influx of neutrophils, eosinophils, monocytes, NK cells, macrophages and DCs at these sites (102, 159). Secretion of type-I and type-III IFNs (including IL-28 and IL-29) is also reported, predominantly via TLR3 signaling (7, 160), suggesting an active protective process against viral infections. Other TLR-dependent mediators of immune responses that are induced in the airways include BAFF and APRIL, which favor interaction of airway epithelial cells with B cells and DCs (161).

### Gut epithelium

The mucosal epithelial tissue of the digestive system, or gut, is tightly connected to both the oral cavity and the respiratory tract. This epithelium is also colonized by an enormous number of commensal microorganisms (approximately 10^13^–10^14^) and occasionally, a small number of pathogens from similar species to those found in the oral and respiratory tracts. Differential TLR expression and functions are observed in epithelial cells of these tissues, depending on the specific location (i.e., the esophagus, the stomach, the small intestine or the large intestine) (Table 1), and depending on the local commensal flora in each of these tracts. Generally, mRNA for TLR1 to TLR9 has been reported, but only a low, constitutive expression of TLR2, TLR4 and TLR5 (and of MD-2) is observed on the cell basolateral side (120).

Few studies have shown TLR expression in the esophagus. TLR4 mRNA expression and functional activity in response to LPS have been shown in biopsies of cancerous and normal esophageal epithelial tissues *in vitro* and *ex vivo* (114). Esophageal epithelial cells also express high levels of TLR2 and TLR3 mRNA and, to a lower extent, TLR1 and TLR5 mRNA. Following stimulation of TLR3 with Poly I:C, TLR2 mRNA expression is up-regulated, although these cells are unresponsive to PG and Pam_3_CSK_4._ In addition, cell incubation with flagellin also fails to induce esophageal epithelial cell activation, suggesting that both TLR2 and TLR5 proteins may not be expressed. Secretion of high levels of IL-8 via TLR3 activation is consistent with responses triggered by infection with intracellular pathogens (113). Only a few studies exist on stomach epithelial cells. In normal gastric epithelial cells, constitutive and inducible expression of TLR2, TLR4 and TLR5 mRNA and protein has been shown in response to bacterial components and whole organisms (115, 116). Inducible expression of TLR4 and TLR2-dependent secretion of cytokines *in vitro* can be inhibited by blocking anti-TLR2 antibodies (117).

The small and large intestine areas present the highest concentration of commensal microorganisms as compared to all the other mucosal epithelial sites. Here, TLR expression and control of signaling pathways is extremely important. Intestinal epithelial cells (IECs) may either lack expression of TLR4, MD-2 and CD14 or, if expressed, TLR4 may be located in intracellular compartments to avoid hyper-responsiveness to LPS from commensal organisms (118, 121, 122). Up-regulation of MD-2 and TLR4 can be induced by high local levels of IFN-γ or TNF-α, which is thought to contribute to chronic colitis associated with Crohn’s disease (120). TLR2 mRNA and low levels of TLR2 protein are expressed in these cells at a sub-apical location and high levels of TLR5 are detected on the cell’s basolateral side, thus sensing flagellin only when microorganisms cross the intestinal epithelial barrier during active invasion (119). TLR9 activation at the apical or basolateral side of IECs may induce secretion of different cytokines, depending on whether NF-κB pathways are triggered (123). Polar expression of TLRs is a mechanism common to the oral, airway and gut epithelia for preventing unnecessary and potentially detrimental inflammatory response to commensal colonizers. Additional control mechanisms for TLR-mediated intestinal cell activation include negative regulation of TLR signaling via Tollip and the single Ig IL-1 receptor-related molecule (SIGRR) (162).

Toll-like receptor-dependent production of α-defensins (hD-5 and hD-6), β-defensins (hBD-1, hBD-2, and hBD-3) and other bactericidal substances has been shown in the gut (163). In contrast to the predominant induction of TLR-dependent anti-microbial products in the oral and airway epithelium, secretion of inflammatory mediators is an important outcome in the gut epithelium. Pro-inflammatory cytokines including IL-1β, IL-7, IL-8, IL-15, and IL-18 drive local recruitment of PMNs and other leukocytes (164, 165), and IL-18 secretion further amplifies IL-2 and INF-γ production, influencing production of mucus and its composition. However, considering that an overly robust inflammatory response is detrimental to the host, gut epithelial cells also produce IL-10 and TGF-β, which play a role in tissue repair processes and re-establish the barrier function of the gut epithelia (166).

The significance of TLR-dependent cytokine production by IECs is also related to the presence of M cells, Paneth cells, and mucus-producing goblet cells within the epithelial tissue. M cells, or membranous epithelial cells, are located above Peyer’s patches and other lymphoid areas and provide an important bridge between epithelial cells and professional immune cells. These cells facilitate antigen sampling and transport through the epithelial layer into lymphoid areas and accelerate both innate and adaptive immune responses (167). M cells express higher levels of TLR4 on the apical surface, in contrast to enterocytes, and provide signals for further activation of immune cells to produce secretory IgA for control of both pathogens and commensals and other mediators of immunity such as BAFF and APRIL (168). Paneth cells, epithelial cells of the small intestine, express TLRs for recognition of pathogens and produce anti-microbial substances (124).

### Genito-urinary tract epithelium

Toll-like receptor expression by epithelial cells of the genito-urinary tract is also fine-tuned to specifically respond only to pathogens, because of the potential large number of commensal organisms in the different compartments of this mucosal site (Table 1). In this epithelium, additional variables need consideration, such as the diversity of cell composition of the reproductive tract epithelium in females vs. males, the nature of the commensal flora and the likelihood of exposure to pathogens for each anatomical section, thus suggesting a tissue-specific immune surveillance. Few studies have been carried out on the human male genital tract (MGT), while more abundant data exist on mouse and rat models (169). TLR expression in the human MGT is generally low. In different sections of the MGT, for example in the epididymis, vas deferens, seminal vesicles and testes, epithelial cells do not appear to express TLR mRNA. In the prostate, intracellular expression of TLR3 and TLR8 is detected and that of TLR9 in the penile urethra, although it is not widespread to all individual cells. *In vitro* studies of primary urethral and prostate cells, epididymal-vas deferens have indicated a potential cell susceptibility to activation by TLR2 ligands. Similar observations have been made in seminal vesicles (although overall TLR expression is not well-studied in these cells) (81, 125–127). One of the reasons for such low TLR-dependent signaling in the MGT also correlates with protection of sperm cells development, which would not benefit from occurring in a pro-inflammatory environment where cells are highly responsive to stimulation via TLRs (despite protective functions against infections) (170). In addition, the commensal microflora burden of the MGT is rather low.

Toll-like receptor expression is better described in epithelial cells of the female genital tract (FGT). In vaginal epithelial cells, TLR1 to TLR6 and TLR9 are expressed, with high levels of TLR2 and TLR4 proteins (129, 130). In epithelial cells of upper FGT regions, TLR4 expression is not fully ascertained (133). It is likely low in the endocervical and ectocervical epithelial tissues (171) but these cells express TLR1, TLR2, and TLR6 and are responsive to TLR2 and TLR5 activation. Other reports indicate expression of mRNA for TLR7, TLR8 (weak), TLR9 and detectable protein levels of TLR3 (extracellular) and TLR9 in these cells (130, 132). In the sterile regions of the FGT, the fallopian tubes and uterus, TLR1, TLR7, TLR8 and TLR9 mRNA is detected, likely due to the sensitivity of these sites to viral infections (130). Uterine epithelial cells express TLR1 to TLR9 but are only susceptible to activation by TLR2, TLR3, TLR5, and to some extent, TLR4 agonists (134). Endometrial epithelial cells express TLR1 to TLR6 and TLR9, but low TLR5 and TLR6 levels are observed in isolated endometrial epithelial cells as compared to the whole tissue (130). In most of the FGT epithelia, TLR10 mRNA is also detected. Clearly, such variable expression of TLRs throughout the FGT supports different responses to potential pathogens and controls local homeostasis in the absence of infection.

Although in part physically distinct from the genital tract epithelium, cells of the urinary tract epithelia (comprising the urethra, bladder, ureters, and the kidneys) have also been shown to express TLRs. TLR2, TLR3, TLR4, TLR5, and TLR9 are expressed in various sites of the urinary tract epithelium (137), but renal epithelial cell lines and primary human proximal tubule cells do not express TLR4 and are unresponsive to LPS (138, 139). Expression of soluble MD-2 and CD14 also correlates with responsiveness of these cells to LPS. In the bladder and in the kidney epithelia, expression of TLR4 is an important surveillance strategy against Gram negative bacteria infections, particularly uropathogenic *E. coli*, controlling inflammatory responses to such infections (140). Heightened susceptibility to urinary tract infections (UTIs), asymptomatic and persistent bacteriuria have been associated to low levels of TLR4 expression and TLR4 polymorphisms in human being (135).

Although there are tissue-specific differences in TLR expression in the MGT and the FGT, production of anti-microbial substances is observed in both tissues. TLR-dependent secretion of hBD-1, DEFB118, DEFB126, and SPAG11 (172) is elicited in the epididymis, testis, and prostate (173) and that of hD-5 and hBD-1 in the vagina, the ectocervix and at high levels particularly in the endocervix, uterus and fallopian tubes. By contrast, different patterns of cytokine and chemokine secretion characterize TLR-dependent responses in the MGT and the FGT, where the delicate balance between homeostasis and inflammation can influence fertility and reproduction processes. In the FGT, mild inflammatory responses are observed, possibly due to an intrinsic bias of this tissue to exposure to large numbers of commensals. TLR-dependent stimulation of FGT epithelial cells *in vitro* induces secretion of IL-1α, IL-1β, IL-6, IL-8 and TNF-α (64, 174), and cyclooxygenase 2 (COX-2), an inducible enzyme associated with mucosal inflammation (131), but few studies exist to support these findings *in vivo*. During infection by sexually transmitted pathogens, such as *Chlamydia* or *Neisseria gonorrhoeae*, secretion of IFN-γ, IL-10, IL-12, IL-1β, IL-6, and IL-8 has been reported in the cervix, fallopian tubes, and cervical secretions (174, 175). In the fallopian tubes and the uterus, the presence of endometrial epithelial cells, with similar functions than the intestinal M cells, favors secretion of inflammatory cytokines that can influence local immune responses (176). In epithelial cells of the MGT, data gathered mostly from *in vitro* studies have identified secretion of IL-6, IL-8, TNF-α, and IL-1β following TLR stimulation (64, 128, 177). A consequence of genito-urinary tract epithelia inflammation, recruitment of professional APCs and PMNs to the site of infection leads to symptoms such as purulent discharge and local tissue inflammation. These symptoms are observed at varying extent in both the MGT and FGT.

## Conclusion

It is well established that cell activation and signaling via TLRs is crucial for induction of host immune and defense responses against microorganisms. During the course of life, human beings encounter a large number of commensal bacteria. The majority of commensals do not alter local homeostasis and integrity of the tissues that they colonize, and some have even beneficial affects at mucosal sites, for example the gut or the reproductive tract. Naturally, host mucosal epithelia are also exposed to potential pathogens. In many cases, these microorganisms only cause disease when they succeed in colonizing the appropriate infection site(s) or in crossing the mucosal epithelial barriers that separate the host from the environment. Potential pathogenicity and diseases may also arise in the event of cross-colonization of mucosal epithelial tissues by commensal organisms that are not specific for that given body site. Thus, epithelial cells of mucosal sites have evolved to implement specific control mechanisms for bacterial recognition and for initiating or suppressing local tissue-specific immune responses against disease-causing organisms or commensals, respectively. Regulation of TLR expression, cellular localization, and functions in mucosal epithelial cells provides one of the mechanisms by which bacteria/host cell interactions are directed to avoid onset of persistent local inflammation. For example, in mucosal epithelia, expression of TLRs on the cell surface is strongly regulated between the apical and basolateral sides of cells, ensuring that immune response only takes place if pathogens cross these tissues and preventing tissue damage in the absence of benefits for the host. Similarly, expression of endosomal TLRs and other cytosolic PRRs including NLRs (165) and RIG-like receptors (RLRs) (178) ensures recognition of intracellular pathogens. Thus, induction of specific responses is targeted to pathogen organisms’ clearance and resolution of infection. The importance of TLR regulation is also apparent in the control of host diseases and conditions that can result from abnormal TLR expression and *tlr* gene polymorphisms. For example, Lupus and other auto-immune conditions have been suggested to be caused, in part, by dysregulation of TLR expression (179). Host susceptibility to infections can also be linked to some TLR polymorphysms, as shown for malaria, uropathogenic *E. coli*, and a number of other bacterial infections (135, 136).

Toll-like receptor expression also can influence specific microbial communities present at mucosal epithelial sites, as shown for the gut or the genito-urinary tract. Although a number of factors likely influence the intimate relationship between host and microbes, TLRs may play a role in determining specific colonization sites for commensal bacterial. The expression and regulation of TLRs in epithelial cells is thus of critical importance for microorganisms sampling and act to direct downstream host responses and, ultimately, disease outcomes.

## Conflict of Interest Statement

The authors declare that the research was conducted in the absence of any commercial or financial relationships that could be construed as a potential conflict of interest.
